# Escaping social rejection, gaining total capital: the complex psychological experience of female genital mutilation/cutting (FGM/C) among the Izzi in Southeast Nigeria

**DOI:** 10.1186/s12978-022-01348-3

**Published:** 2022-02-14

**Authors:** Olayinka Omigbodun, Tolulope Bella-Awusah, Nkechi Emma-Echiegu, Jibril Abdulmalik, Akinyinka Omigbodun, Marie-Hélène Doucet, Danielle Groleau

**Affiliations:** 1 Centre for Child & Adolescent Mental Health & Department of Psychiatry, College of Medicine, University of Ibadan, Ibadan, Nigeria; 2 Department of Psychology and Sociological Studies, Ebonyi State University, Abakaliki, Nigeria; 3 Division of Social and Transcultural Psychiatry, McGill University, Montreal, Canada; 4 Culture and Mental Health Research Unit, Lady Davis Medical Institute, Jewish General Hospital, Montreal, Canada; 5 Department of Obstetrics and Gynaecology, College of Medicine, University of Ibadan Nigeria, Ibadan, Nigeria

**Keywords:** FGM/C, Psychological experience, Nigeria, Global mental health, Power, Capital, Habitus, Adolescents, McGill Illness Narrative Interview Schedule (MINI), MGF/E, expérience psychologique, Nigeria, santé mentale globale, pouvoir, capital, habitus, adolescence, McGill Illness Narrative Interview Schedule (MINI), M/AGF, experiencia psicológica, Nigeria, salud mental global, poder, capital, habitus, adolescencia, McGill Illness Narrative Interview Schedule (MINI), MGF, experiência psicológica, Nigéria, saúde mental global, poder, capital, hábito, adolescência, McGill Illness Narrative Interview Schedule (MINI)

## Abstract

**Background:**

While the deleterious effects of FGM/C on physical health are well documented, the psychological experience of this harmful practice is a neglected area of research, which limits global mental health actions. As FGM/C was a traditional practice in some areas of Nigeria, the study aimed to understand the psychological experience of FGM/C in context.

**Methods:**

This qualitative study was completed in urban and rural Izzi communities in Southeast Nigeria where FGM/C was widely practiced. In-depth interviews were completed with 38 women of the same ethnicity using the McGill Illness Narrative Interview (MINI) to explore the collective psychological experience of FGM/C before, during and after the procedure. The MINI was successfully adapted to explore the meaning and experience of FGM/C. We completed thematic content analysis and used the concepts of total capital and habitus by Bourdieu to interpret the data.

**Results:**

During the period of adolescence, Izzi young women who had not yet undergone FGM/C reported retrospectively being subjected to intense stigma, humiliation and rejection by their cut peers. Alongside the social benefits from FGM/C the ongoing psychological suffering led many to accept or request to be cut, to end their psychological torture. Virtually all women reported symptoms of severe distress before, during and after the procedure. Some expressed the emotion of relief from knowing their psychological torture would end and that they would gain social acceptance and total capital from being cut. Newly cut young women also expressed that they looked forward to harassing and stigmatizing uncut ones, therein engaging in a complex habitus that underscores their severe trauma as well as their newly acquired enhanced social status.

**Conclusion:**

FGM/C is profoundly embedded in the local culture, prevention strategies need to involve the whole community to develop preventive pathways in a participatory way that empowers girls and women while preventing the deleterious psychological effects of FGM/C and corresponding stigma. Results suggest the need to provide psychological support for girls and women of practicing Izzi communities of Southeast Nigeria.

## Plain Language Summary

While the deleterious effects of FGM/C on physical health are well documented, we have little knowledge on the psychological experience of this harmful practice. This study aimed to understand the psychological experience of FGM/C in Izzi communities in Southeast Nigeria where FGM/C was widely practiced. In-depth interviews were completed with 38 women from the same ethnic group to explore the collective psychological experience of FGM/C before, during and after the procedure.

Our results found that during adolescence, Izzi girls who had not yet undergone FGM/C reported being subjected to intense stigma, humiliation and rejection by their cut peers. This ongoing psychological suffering led many young women to accept or request to be cut, in order to end their psychological torture. Virtually all women reported experiencing severe distress before, during and after the procedure. Some expressed relief from knowing their psychological torture would end and that they would gain social acceptance and a rise in status from being cut. Newly cut young women also expressed that they looked forward to harassing and stigmatizing uncut ones, therein engaging in a complex behaviour that underscores their severe trauma as well as their newly acquired enhanced social status. Our results suggest that prevention strategies need to involve the whole community to develop preventive pathways in a participatory way that empowers girls and women while preventing the deleterious psychological effects of FGM/C and corresponding stigmatizing behaviour towards the uncut. Results suggest the need to provide psychological support for girls and women of practicing Izzi communities of Southeast Nigeria.

## Résumé

**Contexte:**

Si les effets néfastes des mutilations génitales féminines/excision (MGF/E) sur la santé physique sont bien documentés, l'expérience psychologique de cette pratique délétère est un domaine de recherche négligé, ce qui limite les actions mondiales en matière de santé mentale. Les MGF/E constituant une pratique traditionnelle dans certaines régions du Nigeria, l'étude visait à comprendre l'expérience psychologique des MGF/E dans son contexte culturel.

**Méthodes:**

Cette étude qualitative a été réalisée au sein de communautés Izzi urbaines et rurales du sud-est du Nigeria où les MGF/E constituent une pratique courante. Des entretiens approfondis ont été réalisés avec 38 femmes de la même ethnie en utilisant le McGill Illness Narrative Interview (MINI) pour explorer l'expérience psychologique collective des MGF/E avant, pendant et après la procédure. Le MINI a été adapté avec succès pour explorer la signification et l'expérience d’une MGF/E. Nous avons effectué une analyse de contenu thématique et utilisé les concepts de capital total et d'habitus de Bourdieu pour interpréter les données.

**Résultats:**

Pendant la période de l'adolescence, les jeunes femmes Izzi qui n'avaient pas encore subi une MGF/E ont rapporté rétrospectivement avoir été soumises à une stigmatisation, une humiliation et un rejet intense par leurs pairs ayant été mutilées. Outre les avantages sociaux d'être excisée, la souffrance psychologique incessante a conduit beaucoup de non excisées à accepter ou à demander d'être excisées, pour mettre fin à leur torture psychologique. Pratiquement toutes les interviewées ont signalé des symptômes de détresse grave avant, pendant et après l'intervention. Certaines ont exprimé un grand soulagement sachant que leur torture psychologique prendrait fin et que l'excision leur apporterait une acceptation sociale et un gain important quant à leur capital total. Les jeunes femmes nouvellement excisées ont également déclaré qu'elles avaient hâte de harceler et de stigmatiser à leur tour, les adolescentes non excisées, s'engageant ainsi dans un habitus complexe qui souligne leur grave traumatisme ainsi que l'amélioration de leur nouveau statut social.

**Conclusion:**

Les MGF/E étant profondément ancrée dans la culture, les stratégies de prévention doivent impliquer l'ensemble de la communauté afin de développer des voies préventives de manière participative qui donnent du pouvoir aux filles et aux femmes tout en prévenant les effets psychologiques délétères des MGF/E et la stigmatisation associée. Les résultats suggèrent la nécessité de fournir un soutien psychologique aux filles et aux femmes des communautés Izzi pratiquantes du sud-est du Nigeria.

## Resumen

**Contexto:**

Aunque los efectos nocivos de la mutilación/ablación genital femenina (M/AGF) sobre la salud física están bien documentados, la experiencia psicológica de esta práctica nociva es un área de investigación descuidada, lo que limita la acción global sobre la salud mental. Dado que la A/MGF es una práctica tradicional en algunas partes de Nigeria, el estudio pretendía comprender la experiencia psicológica de la A/MGF en su contexto cultural.

**Métodos:**

Este estudio cualitativo se llevó a cabo en comunidades urbanas y rurales de Izzi en el sureste de Nigeria, donde la M/AGF es una práctica común. Se realizaron entrevistas en profundidad a 38 mujeres de la misma etnia utilizando la McGill Illness Narrative Interview Schedule (MINI) para explorar la experiencia psicológica colectiva de la M/AGF antes, durante y después del procedimiento. El MINI se adaptó con éxito para explorar el significado y la experiencia de la M/AGF. Realizamos un análisis de contenido temático y utilizamos los conceptos de Bourdieu de capital total y habitus para interpretar los datos.

**Resultados:**

Durante la adolescencia, las jóvenes Izzi que aún no se habían sometido a la M/AGF informaron retrospectivamente de que habían sido sometidas a un intenso estigma, humillación y rechazo por parte de sus compañeras que habían sido mutiladas. Además de los beneficios sociales de la ablación, el incesante sufrimiento psicológico llevó a muchas mujeres no sometidas a la ablación a aceptarla o solicitarla, para poner fin a su tortura psicológica. Casi todos los entrevistados informaron de síntomas de gran angustia antes, durante y después del procedimiento. Algunos expresaron un gran alivio al saber que su tortura psicológica terminaría y que la ablación les traería aceptación social y una ganancia significativa en su patrimonio total. Las jóvenes recién circuncidadas también declararon que esperaban acosar y estigmatizar a su vez a los adolescentes no circuncidados, participando así en un complejo habitus que subrayaba su grave trauma, así como su nueva condición social.

**Conclusión:**

Dado que la A/MGF está profundamente arraigada en la cultura, las estrategias de prevención deben implicar a toda la comunidad para desarrollar vías de prevención de forma participativa que empoderen a las niñas y a las mujeres, al tiempo que evitan los efectos psicológicos nocivos de la A/MGF y el estigma asociado. Los resultados sugieren la necesidad de proporcionar apoyo psicológico a las niñas y mujeres de las comunidades izzi practicantes del sureste de Nigeria.

## Abstrato

**Antecedentes:**

Embora os efeitos prejudiciais da mutilação genital feminina (MGF) sobre a saúde física estejam bem documentados, a experiência psicológica desta prática deletéria é uma área negligenciada de pesquisa, limitando a ação global sobre a saúde mental. Como a MGF é uma prática tradicional em algumas partes da Nigéria, o estudo teve como objetivo compreender a experiência psicológica da MGF em seu contexto cultural.

**Métodos:**

Este estudo qualitativo foi realizado em comunidades Izzi urbanas e rurais no sudeste da Nigéria, onde a MGF é uma prática comum. Foram realizadas entrevistas em profundidade com 38 mulheres da mesma etnia usando a McGill Illness Narrative Interview Schedule (MINI) para explorar a experiência psicológica coletiva da MGF antes, durante e após o procedimento. O MINI foi adaptado com sucesso para explorar o significado e a experiência da MGF. Conduzimos uma análise temática de conteúdo e utilizamos os conceitos de Bourdieu de capital total e hábito para interpretar os dados.

**Resultados:**

Durante a adolescência, jovens mulheres izzi que ainda não haviam sido submetidas à MGF relataram, retrospectivamente, terem sido submetidas a intenso estigma, humilhação e rejeição por parte de seus pares que haviam sido mutiladas. Além dos benefícios sociais de serem cortadas, o sofrimento psicológico incessante levou muitas mulheres não cortadas a aceitar ou a pedir para serem cortadas, para acabar com sua tortura psicológica. Quase todos os entrevistados relataram sintomas de grande sofrimento antes, durante e após o procedimento. Alguns expressaram grande alívio sabendo que sua tortura psicológica terminaria e que o corte lhes traria aceitação social e um ganho significativo em seu patrimônio total. As jovens recém circuncidadas também declararam que esperavam assediar e estigmatizar as adolescentes não circuncidadas por sua vez, envolvendo-se assim em um hábito complexo que sublinhava seu severo trauma, assim como seu novo status social.

**Conclusão:**

Como a MGF está profundamente enraizada na cultura, as estratégias de prevenção devem envolver toda a comunidade a fim de desenvolver caminhos de prevenção de forma participativa que empoderem meninas e mulheres, enquanto previnem os efeitos psicológicos deletérios da MGF e o estigma associado. Os resultados sugerem a necessidade de fornecer apoio psicológico a meninas e mulheres na prática das comunidades izzi no sudeste da Nigéria.

## Introduction

Female genital mutilation/cutting (FGM/C), referred to in many practicing groups as circumcision, comprises all procedures that involve partial or total removal of the female external genitalia and/or injury to the female genital organs for cultural or any other non-therapeutic reasons [1, 2].

While the physical health consequences of FGM/C are well documented, studies on the psychological experience and consequences of FGM/C are limited both in number in scope and in quality [1, 3–6]. According to the latest reviews [1, 3, 7] FGM/C is likely to produce adverse mental health outcomes such as anxiety, somatisation, phobias, low self-esteem, depression and other affective disorders and a higher likelihood of psychiatric disorders such as PTSD in those who undergo the procedure. However, the design of these studies did not provide enough evidence to draw conclusions on the experience and corresponding psychosocial consequences of FGM/C. The psychological experience and psychopathology surrounding FGM/C is thus a neglected area in the FGM/C literature which limits guidance for global mental health policy and programming that aims to eradicate, prevent and provide psychological counselling relating to FGM/C in practicing as well as host countries with immigrants and refugees from practicing countries. There is thus an urgent need to better understand the complexity of the psychological experience of FGM/C in social and cultural context.

### Background of the study

Nigeria has the highest absolute number of cases of FGM/C in the world, harbouring a quarter of the world’s estimated 115–130 million cut/circumcised girls and women [8]. With the signing of the Violence Against Persons (Prohibition) Act of 2015 into law in Nigeria, FGM/C is now a criminal offence in Nigeria, as all forms of violence against persons are prohibited [9].

While the current national prevalence rate of FGM/C among adult women in Nigeria is 20% the country is well known for the wide variation of prevalence across states, ethnic groups, and regions [10]. For example, the South East (35%) and the South West (30%) of the country present with the highest prevalence rates of FGM/C among adult women, while the North East region (6%) presents with the lowest rate in the country [11]. The prevalence of FGM/C also varies by state with 62% percent of women in the Imo state having experienced FGM, compared with less than 1% of those living in Adamawa and Gombe states [11]. In the Ebonyi State (51–62%) where we did or study, the prevalence of FGM/C presents among the highest in the country [11]. FGM/C is performed at different ages in Nigeria, however the majority of circumcised women (86%) (age 15–49) were cut before the age of 5, while only 5% were circumcised at an older age usually at 15 or older [11]. The Igbo of the South East of Nigeria are known to practice FGM/C later in adolescence and sometimes before the birth of the first child [10]. According to recent data the Izzi sub-tribe of the Igbo ethnic group, where we did our study, also seem to present with a raise in the practice of FGM/C with women born more recently (between 1999 and 2003) presenting with a higher rate of FGM (87%) compared to those born earlier (1959–1963) associated with a lower rate (73.9%) [10].

Studies done in these regions reveal a strong cultural support for the persistence of the practice, with various reasons such as chastity, fidelity, marriageability, transition to womanhood, rise in status and tradition being adduced to justify the practice [11–13]. The Izzi sub-tribe of the Igbo ethnic group, located in Ebonyi State in Southeast Nigeria, was selected for this study because all types of FGM/C are performed, with 46% being types I and II, 19% type III with infibulations and 33% of indeterminate type [14].

The chosen urban site for the study was Amike Aba Community, in Abakaliki, the state capital and major urban settlement of Ebonyi State. The chosen rural site was the Mgbalukwu Community in Izzi Local Government Area. Among the Izzi, FGM/C is locally called circumcision and is described as a compulsory rite of initiation and the only avenue through which an Izzi woman could be allowed matrimonial rights, to participate in community affairs, enter full womanhood and bear children considered to be normal [14]. This practice occurs during adolescence or early adulthood among the Izzi, unlike in other ethnic groups were FGM/C is carried out in infancy or early childhood [10]. Carrying out the study in a community where FGM/C is done during adolescence provided the opportunity to obtain information on the psychological experiences of being cut as well as the period before and after the procedure. These characteristics led to the selection of the study sites. The research question that guided this study was, “what was the psychological experiences surrounding FGM/C in women across the life cycle in the Izzi rural and urban communities of Southeast Nigeria”. This article is the second in a series of studies in which qualitative methods are used to determine the psychological experiences of FGM/C through the life cycle [5].

## Methods

### Sample and sampling

The sample for this study comprised 38 young and older women who have undergone the FGM/C procedure with 20 living in urban settings and 18 in rural communities of Ebonyi state, Southeast Nigeria. Using the snowball sampling technique participants were initially identified as having been cut by community members and invited to participate in an interview [15]. Given that the psychological experience of FGM is a difficult subject to discuss with a stranger, we choose snowball sampling in order to facilitate communication and foster trust. As well, given the financial restrictions of the research, the snowball sampling offered the practical advantage of reaching the target population in a timely manner. We aimed to attain as much sociodemographic variation in the sample as possible to enhance theoretical generalizability, which is why we recruited women from urban and rural areas, and varied level of age and education.

#### Sample characteristics

**Table:** 

Characteristic	Number
Sample size	38
Age (years)	
18–19	1
20–29	8
30–39	7
40–49	10
50–59	4
60 and above	1
Unknown	6
Location	
Rural	18
Urban	20

### McGill Illness Narrative Interview (MINI)

We aimed to explore the women’s collective dimension of the embodied and emotional experience of FGM/C in the Izzi cultural context. Based on the premise that FGM/C generates a bodily injury that involves a treatment and healing period, the McGill Illness Narrative Interview Schedule (MINI) was chosen as it is conceptualized to explore the complexity of the psychological and embodied experience of health problems in a cultural context [16]. The initial English generic version of the MINI was adapted by the last author (DG) to explore the experience of FGM/C and was thereafter translated into the Izzi dialect by one of interviewers with a bachelor degree and fluent in the Izzi dialect of Igbo and an expert in the Izzi dialect and English Language did the back translation (see Appendix for the English version of the MINI adapted for FGM/C). The entire team of researchers then met to finalise the instrument, which was then pretested in the Izzi speaking communities and adjusted for clarity. The four main sections of the MINI [16] were adapted to explore the FGM/C experience. The first section seeks to elicit a narrative of the sequence of events leading to the psychological and embodied experience of FGM/C in the cultural context. The second section aims to ask the interviewee to identify and compare one’s FGM/C experience with prototypical experiences of FGM/C involving family members, community members and the media. The third section explores the lay explanatory models the interviewee uses to explain why they underwent FGM/C as well as local labels used to refer to the experience. The fourth section explores pathways to care following FGM/C, potential issues with treatment, and life changes brought about by the FGM/C.^1^

### Interview process

Each interview lasted an average of 1–2 h and was conducted in a quiet, conducive place within the community, usually under a tree, in the town hall or a clearing in the bush. We ensured that the interviews took place out of earshot of others, such that while others could see from a distance, they definitely could not hear the conversation taking place to ensure confidentiality. Before the interview process started, interviewees were informed that the questions would focus on their personal psychological experience of FGM and that they may find the process difficult at some point and could stop whenever they wanted. Any participant that felt distraught during the process was asked if they would like to stop the interview with empathy, and offered a referral to the team psychologist if needed. While some interviewees did pause while narrating difficult segments of their story, none chose to stop their interview completely or wished to meet with the team psychologist.

Each participant received 500 Nigerian Naira (3US Dollars) to cover the cost of refreshments.

The third author of this paper (NE) completed 14 of the 38 interviews, while the remaining 24 were carried out by 5 research assistants who were university graduates who could speak and understand the Izzi dialect. Three of the interviewers were women and two were men. While the team was concerned about the possibility that part of the FGM experience would not be disclosed to a male interviewer, they rapidly were reassured after comparing the transcriptions of the interviews done by female interviewers. In addition, the male interviewers had previous experience in conducting field research in the area under study. One of the co-authors (NE), acting as the fieldwork leader for the study, also indicated that it was best to have a mix of men and women to carry out the interviews as the cutting process was carried out by both.

### Data analysis

The interviews conducted with the adapted MINI were tape-recorded and transcribed in Izzi dialect. NE also listened to the tapes and cross-checked with the Izzi transcription to ensure that it corresponded with the audio recording and also ensured that key ideas and quotes were well captured. All the other team members read through the transcriptions and raised queries, which were resolved after discussion. The Izzi transcriptions were thereafter translated into English and transcribed a second time. Following the transcription of the interviews, the principal investigator (OO) and two co-investigators (TB and JA) held a 3-day data analysis workshop in Ibadan (Southwest Nigeria) to read through the transcribed interviews, identify ambiguous sentences, phrases and words, and seek clarification from the research team in Abakaliki where the interviews were conducted. Another qualitative analysis workshop led by the senior author (DG) took place with the research leaders of the team (OO, TB, and JA). Transcripts of the interviews translated in English were used during the workshop and we followed the classic steps of thematic content analysis: (1) familiarization with the data; (2) identifying codes and themes; (3) coding the data set; (4) organizing codes and themes into larger categories of themes [15]. After the initial familiarization with the data (step 1), thematic content analysis was conducted using inductive coding to identify emerging themes (steps 2 & 3). Following this analysis workshop, 14 additional interviews (7 urban and 7 rural) were completed to ensure data saturation [15], for a total of 38 final interviews. The remaining interviews were transcribed and coded. We therefore organized the thematic codes into larger thematic category of codes for which we produced summaries. These summaries allowed us to identify psychological experiences as they occurred before, during, and after undergoing FGM/C. Distinguishing psychological experience according to this temporality allowed us to both contextualize the complexity of the experience and identify its variation over time. Our conceptual framework was thereafter used to interpret the summaries of the codes in the objective of answering the research question.

### Theoretical framework

In this study, we build from Bourdieu’s [17, 18] concepts of habitus and total capital to guide our interpretation of the coded transcripts of the narrative relating to the psychological experiences of FGM/C. We choose to use the above-mentioned concepts as a lens to understanding the complexity of the overall experience of FGM/C asking if and how the psychological dimension of the experience is governed by corresponding gains or loss of power before, during and after being cut. Bourdieu states that in any social space individuals find themselves in power relations with others and that access to power is determined by total capital which includes economic, social, cultural and symbolic forms of capitals [19–21]. Of relevance to the interpretation of our results, social capital refers to networks of social connections that can be called upon for help and support; economic capital refers to material assets that are convertible into money or property rights; cultural capital can occur in different forms with one being the embodied state corresponding to “long-lasting dispositions of the mind and body” [17, 18] expressed in a person’s means of communication and self-presentation, acquired from their own cultural background [22]. Bourdieu defines symbolic capital as the prestige and respect a person has acquired based on specific accomplishments and/or status that are valued in specific social spaces. Finally, Bourdieu [18] defines habitus as a mental disposition, inculcated by the familial and social environment, that constitutes a way of being and using the body that feels natural for the person and close ones. Considering that FGM/C is not a private practice but a cultural rite of passage to womanhood inscribed onto the body, we also examined, for the purpose of examining the changing potential of this practice, if and to what extent, the practice of FGM/C constituted a habitus. In the logic of the feasibility of developing culturally appropriate strategies to eradicate FGM/C, the question of FGM/C being or not a habitus, is key to guide our reflections on the relevancy of future actions.

## Results

We recruited a total of 38 women including 17 women from rural areas between the ages of 18–58 years old and 21 women living in urban areas from the ages of 21–60 years. Participants of this study produced narratives relating to their psychological experience of FGM/C which they structured around five temporal phases including: (1) the ‘akpapyi’ period characterized by socio-emotional suffering; (2) the emotional elevation phase following the decision to be cut; (3) the cutting procedure where girls experiences ‘flight or fight’ response and extreme pain; (4) the post-cutting period characterized by mixed emotions including elevation, trauma and betrayal and; (5) the long term period where they spoke of the psychological consequences of FGM/C.

1. The ‘akpapyi’ period. The emotional experience relating to the ‘akpapyi’ period is key to understanding why young women accept, or even request in some cases, to undergo the FGM/C procedure. It is a pejorative term used to describe the ‘uncircumcised’ women. Virtually all participants described experiencing intense negative psychological feelings during this period of their lives ranging from anger, sadness, shame and embarrassment. In some instances, these emotions were accompanied by social withdrawal with the community exhibiting extreme forms of stigma toward uncut women and girls. Furthermore, this community did not allow the akpapyi to gather with the ‘circumcised’ and if they approached such gatherings, they were asked to leave. Women across the life cycle in urban and rural areas recounted feelings of shame and anger recalling being mocked and humiliated during their ‘akpapyi’ period.
  Before I went for circumcision, I used to be ashamed of the insults received from people. As they mocked me, it annoyed me. Why it annoys me is because they mock me saying I’m an uncircumcised (akpapyi) that I should look at my size. (45 years, urban)
  The stigma against the akpapyi is sustained by a community discourse stating that the uncut behave badly, have increased libido (which is perceived negatively), are promiscuous and are likely to have adverse experiences when they give birth. This stigmatization also extends to their children as they are often portrayed as being considered less than human.
  If I married without circumcision and eventually gave birth to a child or children, the child or children cannot be taken as a human being and are described as fools. (47 years, rural)
  I heard that a pregnant woman…. in the process of her delivery, met some women to help her to deliver, when they opened her wrapper and saw her private part (she was uncut), they abandoned her to go and invite others to come and see the horrible thing they are seeing (45 years, rural)
  Participant recalled that while they were an ‘akpapyi’ they responded with strong feelings of sadness, crying, feeling unaccepted and social withdrawal, depressive symptoms which included dropping out of school for some when they were still girls. Subsequently, many participants felt resignation toward the inevitability of undergoing FGM/C while putting aside their initial fear or apprehension of the procedure. Escaping the experience of extreme stigma during the ‘akpapyi’ period gave rise to a strong justification for undergoing FGM/C. Many describe the determination to do the FGM/C as a bid to avoid mocking and insults, remove the shame, humiliation and isolation, and be able and comfortable to participate in society.
  I have already volunteered myself for the cutting because of the insult of being called “akpapyi” and if you did not circumcise, it was not good then. (48 years: urban)
  When we are bathing together, my mates will be laughing at me and I was ashamed and I told my mother that I will be going for circumcision. (34 years, rural)
  Being among their peers and being allowed to participate in social activities was retrospectively particularly important for them as younger girls. They described the need to be ‘among’ or ‘belong’ as a reason for deciding to go for the FGM/C.
2. The decision to be cut: emotional elevation. Some of the women recounted experiencing emotional elevation once the decision to be cut was made and as they were approaching the date of their cutting. The sense of happy anticipation was clearly evident as they approached the ‘circumcision’ day and as plans were made for the event.
  I was very happy. I was even the first person to wake up and reminded my mother: “hope you said I will be circumcised today” (25 years, urban)
  The anticipation of happiness was associated with escaping the akpapyi experience and what they described as great joy appears in many instances to be due to a sense of liberation and relief as seen in several of the narratives of our participants.
  I was happy when they told me that I was going to undergo circumcision because I have been embarrassed enough. (19 years, urban)
  The way it was to me is that I’m unhappy because any time I go out, my mates will be mocking me. One day, I told my mother how my mates had been molesting me about being uncircumcised and I asked her to let me be circumcised. She accepted and after a while she asked me to prepare that she will take me to the place I will be circumcised, and I was happy. (30 years, rural)
  My age mates insulted me so much by calling me names like “akpapyi” any time I am in their midst and I used to feel so bad, so when I had the chance to have it performed on me, I was so happy and ran to the woman’s house to have it done. My family members were not even at home when I ran to the woman’s house with my friends to have it done! (20 years, rural)
  Narratives also revealed that this feeling of happiness was also associated with the clear rise in social status provided by the cutting which was seen as a rite of passage to womanhood and respectability.
  What I saw then that made me feel happy was that since the circumcision can make me complete as tradition prescribed, I said let me do it just as my mates are doing it. Just as you know that there is a difference between a girl and a woman, and the beginning of womanhood starts with this circumcision. That is why I was glad to do it as this will lead me into womanhood. (46 years, rural)
3. During the FGM/C procedure: ‘fight or flight’ response. As they stood in queue waiting for their own turn to be cut, many expressed intense fear from hearing the shouts of those who had just gone through the procedure.
  Before the circumcision, I was afraid and shivering because I heard the cry of the first person, and I started shaking because I did not know how mine will be. (28 years, rural)
  Whether waiting in line or during the actual cutting of their genitals many interviewees described experiencing an acute physiological response of ‘fight or flight’ in reaction to the extreme stress and fear imposed by the imminent danger and pain they experienced. They explained experiencing fear and immense anxiety, palpitations, and trying to run away from the terror of being cut. They knew this procedure could cause serious complications such as pain, bleeding or ultimately death. Their narratives report that in response to the fear exhibited by the young women, the cutters commonly used aggression to obtain their compliance.
  When I saw others cry and bleed during their circumcision, I was terrified. And when it was my turn, and I was held hands and legs down, I was terribly frightened. (20 years, rural)
  Reports of crying and shouting during the procedure were common in the narratives and the reactions of the cutters to this reaction varied with either leaving those to be cut for a while or getting strong men to hold them down and in some cases, sitting on the young women during the procedure to keep them still.
  It was paining me and due to the pains and cry, the woman that was giving the cut now started feeling for me and said they should leave me and continue with others. After theirs, she will now finish my own. (26 years, urban)
  If you are the type of person that does not know how to sit or may cry, someone will sit on your chest, a strong man and he will hold your two hands so that you will not be shaking your bottom. (60 years, urban)
  The first people that were circumcised laid down but I was so much afraid that I was held by people in a sitting and leaning position and they held my two legs. (34 years, rural)
  Adding to the fear of physical complications and potential death resulting from the procedure, participants mentioned also fearing the stigma imposed on those who experienced complications following the actual cutting. Some participants mentioned that the stigma against those who experienced complications, prevents them from having access to those awaiting to be circumcised because it was believed that complications were ‘contagious’ and would be transmitted.
  Many women also reported that they were told that crying and shouting means weakness.
  What actually happened was that during the cutting, if you eventually cry and people were asked to hold you, it means that you are not strong and will be ashamed of yourself just like during my own circumcision; so because of that, people don’t tell the story about it. (47 years, rural)
  Possibly due to flashbacks from the cutting event, evidence of distress was for example recorded in a 28 year old rural dweller as she took deep breaths and paused when describing the event which in her own words ‘tore her body apart’.
4. Emotional states after the cutting. Our participants expressed a variety of different emotions and feelings after the cutting was completed. These varied from having mixed feelings, betrayal, anger, fear of dying from the extreme pain or blood loss, but also feeling of happiness from escaping stigma and gaining a higher status that conveyed respect and their reintegration into the community.
  I became happy because I hate being called ‘akpapyi’, it put an end to insults. (34 years, rural)
  I was so glad to have done it because that brings about the end of the insults I used to receive, with a welcome into the community. (45 years, urban).
  I can’t really say but the only thing I know is that after the circumcision, I was so happy that each time I see my friends I will tell them that I have done the circumcision. Because I am no longer akpapyi, and my friends will not be abusing me again.
  During the procedure it was painful and after the procedure I was happy because I can go to any place, no more embarrassment and partake in events with my age mates. (55 years, rural).
  Some of the things that brought on joy and happiness were also the change that came with FGM/C that made them thereafter eligible for marriage or it meant they would be receiving special attention from family and friends such as having a special meal cooked for them. Many also expressed being happy because of their desire to participate in mocking the other ‘akpapyis’, a severe stigmatizing situation from which they had just recently escaped from.
  Ah! It sweets my mind (made me very happy), because I know that I will follow and make mockery of the others. (45 years, rural).
  Others described a period of mixed feelings in the immediate aftermath of FGM/C. One described it as neither happy nor sad and others as mixed feelings.
  I was not happy again, but with time I ignored the pains. I can’t say that am sad but I was not happy because of the pains. (26 years, urban)
  My feeling then was mixed up. (58 years, rural)
  Moreover, some were afraid of dying because of the extreme pain or important blood loss they experienced. Others may have had organic mental health manifestations due to excessive blood loss, with what appears like clouding of consciousness and fainting.
  After the cutting, it bled so heavily that I nearly died and for 2 days I did not get myself (overwhelmed). The bleeding was so heavy that all my energy was lost and I nearly fainted, until the end of that day I did not get myself. (48 years, urban).
  (Hmmm) yes, I was filled with regrets and lamenting that is this how my life will end just because of circumcision. I was afraid because then I used to have constant dizziness thinking that I have lost all my blood that I will soon die. (30 years, urban).
  It was so painful and I experienced much bleeding, I never knew I will live to survive that day. I felt bad about it especially when I got home; after the procedure I still couldn’t get myself and thinking if I could survive it or not. (45 years, urban).
  Feelings of betrayal by a loved one was expressed by a few who had been lied to about the procedure. Others expressed anger and regret about doing the FGM/C because of the weakness, pain and blood loss they had experienced.
  Hei! My body was so weak that I regretted doing it and I was so angry for doing it. (48 years, urban).
5. Long Term Psychological Consequences of FGM/C. The narratives reveal two distinct psychological long-term responses to undergoing FGM/C. Some expressed emotional elevation from not experiencing any complications following the physical healing period and others experienced clear emotional turmoil suggesting symptoms of PTSD.
  Emotional Elevation in the Absence of Complications. A feeling of happiness was described by some because they had survived the procedure and the FGM/C procedure was over and done, suggesting a sense of relief.
  After the circumcision, I was so happy because I know that am not going to undergo the circumcision again. (26 years, urban).
  I have heard because when they were announcing things they said some people died due to excessive bleeding while some were rushed to the hospital but I am happy because all these things did not occur in my own situation. (25 years, urban).
  Other women experienced long term emotional turmoil after undergoing FGM/C with experiences of flashbacks, symptoms of anxiety and depression, a situation suggestive of Post-traumatic Stress Disorder (PTSD).
  What I remember is that, it is usually any moment I...it is usually any moment I see razor now, any moment I see...razor or scissors, I will remember....and it is what made me now, I don’t use razor to cut nails because, if I see a razor, I will remember when I was circumcised, how it did to my body. That whenever I see razor or scissors, I will remember that circumcision I cut that time. And it is usually when a person has accident, he has wound, that he is sewn something or that....they put scissor to his body or razor, my heart will run to...when they circumcised me, that...that thing was what touched my body (something that has affected me or deeply touched me), it boiled me in the body. (22 years, rural).
  Anger, grief and regret were expressed by a 48 years old woman, whose wound had sealed and who subsequently had difficulty in childbirth.
  After the circumcision was done, the place sealed back, I really suffered as I told you that my body was torn before I was able to deliver my baby as there was no hospital then. Whenever I remember that day I was circumcised, I do get angry and grief and I don’t know whether it is because of the circumcision I did that I don’t use to find it easy when I am pregnant and during child delivery. If I had known I wouldn’t have even thought of it let alone doing it. Even now I cannot take my children for that. It is a regretful act. (48 years, urban)

## Discussion

The results of this study provides insight into the complexity of the psychological experience of FGM/C among Izzi women within their socio-cultural context [23–25]. Considering FGM/C is linked to specific ethnic groups in Nigeria and that a national survey indicates that 1/3 of women are in favor of this practice [26], we will discuss the two psychological processes involved in the willingness of undergoing FGM/C among the Izzi young women while they are aware of the extreme physical pain involved in the practice.

In an African cultural context where identity is more collective than individual and social ties are based on inter-dependency rather than on independence [27, 28], we will argue that the positive emotions of elevation and happiness mentioned in the narratives of our participants are linked to knowing that being cut will both stop the stigma they are being subjected to while providing them with important gains—in what Bourdieu calls total capital—provided by their rise in social status, access to womanhood, respectability, respect of tradition as well as material gains offered during the traditional FGM/C celebration. We will thus discuss below the relations between FGM/C, loss and gains of various forms of capital and how this translates into the complex psychological experience of FGM and corresponding implications for culturally appropriate prevention of FGM/C.

We will thus discuss below the relations between FGM/C, loss and gains of various forms of capital and how this translates into the complex psychological experience of FGM and corresponding implications for culturally appropriate prevention of FGM/C. We will also address the question of habitus by arguing that while the practice of FGM/C is ritualized it is not an habitus. Rather, it is the behaviour of harassing and stigmatizing other non-cut young women that occurs in the aftermath of the FGM/C that constitute a habitus.

### Accepting FGM/C to end psychological torture

The present study reveals that Izzi young women were strongly ostracized and bullied if they were known to be uncut. This very strong stigma and persistence at inflicting shame associated with the Akpapyi status is also described in other cultural groups practicing FGM/C [26, 29, 30]. For example, in the Maasai of East Africa, uncut women with children would not be called a mother, until they were cut [4, 31]. Our results also suggest that the extreme ostracizing and harassment imposed on uncut Izzi young women equates to their social death, leading to great distress, as expressed by their feelings of anger, sadness, shame, embarrassment, social withdrawal and depressive symptoms. They therefore come to resign themselves to be mutilated because they prefer to suffer an acute and major physical pain from a potentially life-threatening procedure than to continue experiencing the unbearable ongoing psychological torture afflicting them. Our results thus suggest that, in the cases where Izzi young women express “happiness” for being cut, this feeling of happiness streams from the relief of knowing their ongoing psychological torture will end followed by a gain in total capital including their social, symbolic, cultural and economic capitals.

### Being cut as a gain in various forms of capitals

The findings of this study reveal that the quality of our participant’s peer social network, what Bourdieu calls their social capital, is highly dependent on the fact of being cut or uncut. This needs to be understood in a context where FGM/C is being practiced by a collectivist society, where social relations are weaved in a web of interdependent relations [23, 28, 32, 33] wherein rites of passage, such as FGM/C [26] contribute to the construction of one’s identity. In a context where people from collectivist groups tend to pay less attention to internal processes and more to external processes to determine their social behavior, it becomes virtually unconceivable to go against such a strong sociocultural norms as FGM/C [34]. Those who attempt to go against these norms suffer deleterious consequences, as the group may ultimately exclude dissenting individuals [18, 35] which is what the participants mentioned was being done to them until they accepted to undergo FGM/C.

Thus FGM/C not only marks the end of the akpapyi period of severe stigma but also provides the young women with a strong reintegration into their peer group, providing them with an identification that is crucial to the construction of their collectivist self [35]. The narratives reveal their need to belong is a powerful, fundamental, and extremely pervasive driving force [36] behind their acceptance of FGM/C. It is therefore not surprising that social acceptance is the reason most frequently mentioned by young women to justify the perpetuation of the FGM/C practice in Nigeria, a phenomenon also observed in other African countries [24, 26, 37].

Young women are also aware they will gain in symbolic capital as soon as they get cut, with immediately gains in prestige, dignity and recognition by becoming officially a women fit to marry. This entry into womanhood confers respect with their new status being celebrated with gifts and food received during the ritualized festivities of the post-FGM/C period, bringing a feeling of happiness mentioned by the participants of our study. This gain in symbolic capital needs to be understood in the context of the central cultural value attributed to women’s reproductive role and maternal body in many African groups [38].

Our results also suggest that FGM/C attributes gains in cultural capital, by creating a maternal body that is thereafter believed to correspond to the culturally valued body of a complete woman, considered to be in control of her sexuality, capable of reproduction and giving birth to “normal” children. Finally, participants mentioned receiving immediate material rewards after being cut, such as gifts and special meals, which also suggest that being cut, will provide gains in economic capital. But more importantly, by becoming eligible for marriage provides cut young women with financial security otherwise hard to obtain. This phenomenon was observed in many other African communities, since a majority of women have neither access to education nor socio-economic conditions to be financially autonomous. Thus “marriageability” through being cut is considered synonymous of survival [23, 33], especially in a context of widespread poverty. Thus, our results suggest that as long as the subsistence of women will be circumscribed within the context of gender inequities and economic dependence on men, and that women will not have access to the empowerment required to be financially independent, young women are likely to conform to the cultural norm for fear of jeopardizing their livelihoods [34].

There is however a relatively recent trend among African men towards wanting FGM/C to end [26] with an increasing number of them, particularly younger and more educated men, being aware of the risks of FGM/C, that would rather marry a non-mutilated woman [25]. Yet one third of Nigerian girls and women report not being aware of what men may think of FGM/C [26]. This may be explained by the fact that FGM/C is virtually never discussed between men and women [26].

### Happiness of passing from victim to persecutor

Above all, a striking finding of this study is the expressed desire and happiness of many newly cut young women to mock and persecute uncircumcised ones. We propose that two related theories, one sociological and the other psychological may explain this phenomenon. We hypothese that by witnessing the perpetuator’s behaviour over a long period of time, Izzi girls and young women unconsciously internalize the fact that the persecuting behaviour is normal and expected from cut young women, a phenomenon Bourdieu calls an habitus; a behaviour that is experienced as natural, normal and expected way of behaving by the ones adopting it and by others of the same group [18]. Thus by persecuting uncut ones, newly cut young women adopt an habitus that is also an expression and affirmation of their newly acquired status and corresponding gain in total capital. Correspondingly, from a psychological perspective, one would think that after being victims of psychological torture and the physical aggression of FGM/C, young women would advocate to protect their uncut peers. In fact, studies have shown, that this type of behaviour of victim becoming aggressor can occur in cases of persistent aggressions exceeding the person’s tolerance, a phenomenon known as the «identification with the aggressor» where a victim experiencing overwhelming fear and anxiety, often during a life-threatening experience, becomes executioner, thereby substituting a passive role to an active one, in which the aggressive revenge provides some form of relief [39, 40]. Moreover, adolescents, in general, who have undergone trauma often re-enact or replicate past traumas in their daily lives [41], which may explain why newly cut young women often display aggressive stigma toward uncut ones.

Paradoxically, this thirst for vengeance is not expressed towards the aggressor: victims are aggressive towards “a substitute” [40], seeking to belong to the group that formerly oppressed them [42, 43]. This reaction would in fact derive from a complex cognitive strategy allowing the victim to cope with the trauma, “to achieve some feeling of strength in an otherwise humiliating situation” [44]. This dehumanized reaction, in which “emotions become detached from the events and dissociation serves as a means of survival” [42], would be stronger when victims have been traumatized over a prolonged period of time [44, 45]. In summary, we hypothese that taking revenge on their uncut peers could be allowing them to make sense of being cut, reduce their cognitive dissonance, as well as find some form of relief from the psychological torture and trauma they experienced while expressing their new status and corresponding social power. But these theoretical interpretations need to be confirmed by future research.

### Negative psychological consequences of FGM/C

The narratives of our participants thus reflect the incommensurable intensity of the psychological and physical aggression and humiliation Izzi young women are subjected both during the Akpapyi phase, as well as before, during and after the FGM/C procedure. These deleterious psychological consequences are, among other things, expressed by the newly adopted behavior of perpetuator and by the fact that many Izzi young women have reported suffering from psychological symptoms such as flashbacks, anxiety and depressive mood even years after the events, which can be related to a post-traumatic stress disorder. Our study also provided insight regarding the underlying psychosocial processes which contribute to the high rates of anxiety, depression and PTSD documented in adolescents who have undergone FGM/C [1, 6, 7, 46].

### Public health implications

The psychological results of this study suggest that knowing about the negative implications of FGM is not enough to prevent young women from voluntarily undergoing FGM/C. Efforts in achieving the eradication of FGM/C should account for the complexity of its underlying psychological, cultural and social intricacies. First, any activity aimed at preventing FGM/C must include mechanisms to eliminate the stigmatization and harassment towards unmutilated young women in the Akpapyi phase. This implies raising awareness—to all members of the community, including cut young women, about the deleterious physical and psychological implications of FGM, including during the uncut period. This also implies finding innovative and culturally appropriate strategies to replace the habitus in which cut young women harass and reject their uncut peers. Other beliefs such as “all men prefer having a cut wife” need an innovative intervention to debunk involving boys and men and key leaders in these communities.

Second, activities aimed at the elimination of FGM/C must offer culturally tailored strategies allowing the provision of new avenues for girls and young women to increase their total capital. For example, finding alternative rites of passage [30] into womanhood could provide young women with symbolic and cultural capital. Other markers of passage into adulthood which could evolve from these communities through discussion and suggestion include reaching a certain level of education [5] which could also contribute to enhancing their economic and symbolic capitals. Finding local strategies such as micro-financing and entrepreneurship for reducing the economic dependency of women for subsistence would also help to enhance their economic capital. Alternative strategies to favor girls’ cultural capital should also be discussed and proposed, such as ways of redefining what a normal and valued body is.

Third, our results suggest, in line with other studies [26], that developing and implementing prevention strategies should involve all members of the community including men and women, young and old as well as cut and uncut young women to better reflect the community dimension of FGM/C. Communities must be empowered to find their own solutions.

Strategies combining education and social mobilization have been shown successful at significantly decreasing the FGM/C prevalence. Among these, Diop and Askew [47] have evaluated a community education program implemented in Senegal, which included not only empowering women, but also involving the whole community towards eliminating FGM/C “through a broad range of educational and health-promoting activities”. The program included components such as education on human rights regarding health and bodily integrity for adults and children, on problem-solving skills contributing to reinforce human rights protection, on the harmful effects of FGM/C on women’s health, including reproductive health. Last but not least, the rejection and social stigma young women experience before undergoing FGM/C is to be taken seriously, because it can gives rise to major deleterious psychological impact. Therefore, prevention of FGM/C program must provide psychological support and counselling for uncut girls and young women if and when they are exposed to harassment and stigma. Furthermore, cut young women need to receive the appropriate care, either from the healthcare system or from community agencies, in accordance with the “World Health Organization guidelines on the management of health complications from FGM/C” [48].

## Limitations of the study

Although the present findings provide socio-culturally contextualized and novel insights into the experience of FGM/C, it has some limitations. Firstly, it was focused on the Izzi ethnic group, which could possibly demonstrate unique behaviours, unshared by other communities. More research is needed in order to find out if this phenomenon of psychological torture is spread among other communities.

Given that women participating in individual interviews were asked to reflect on their past subjective psychological experience leading to, during and after FGM, one needs to keep in mind that their narrative was co-constructed with the interviewer. The data presented here is thus an account of their past subjective experience, not a direct representation of their actual experience. Participants may also have interpreted and reconstructed their past psychological experience through the lens of the conversational context of the interview. While the results of this study must be taken with caution, given that we attained saturation of data, we are confident that it is credible in the sense that it represents what they perceived at the moment of the interview. However, as per any novelty research data, it needs to be validated by future research.

This study was also limited by the fact that only cut women -young and older- were interviewed. It would have been more complete to also interview uncut women and mothers, to find out if they too are pressured to have their daughters cut, as was found in other contexts [37] and younger and older men to understand how they perceived FGM/C in relation to the notion of marriageability. Also, despite the fact that the snowball sampling method that we used is very popular among qualitative researchers it may also have led to a selection bias in our sample. Moreover, we analysed the sample as a whole and did not compare narratives based on age, geography or education levels, variables identified in other African countries as well as Nigeria to influence decision to undergo FGM/C [31]. Future research is thus needed to explore how these variables influence the psychological experience of FGM/C in the context of the changing prevalence rates in the study region.

Finally, more research in different contexts is needed to better apprehend the complex phenomenon surrounding psychological torture around FGM/C.

## Conclusion

The process of undergoing FGM/C involves tremendous suffering and corresponding short and long term deleterious psychological consequences in spite of the emotion of relief expressed by participants when they knew they would escape from social rejection associated with being uncut. This study informs public health program planners and policy makers to address loss and gain in total capital, as well as planning the involvement of whole communities in the process of developing strategies from within to eradicate FGM/C. Eliminating FGM/C is a human rights imperative to protect girls’ and women’s physical, mental and social wellbeing, their bodily integrity and their life, but this needs to be done by taking into account the psychological, social and cultural contexts in which this harmful tradition is being practiced.

## Data Availability

Given the qualitative and personal nature of the data, the data is not available for others outside the research team.

## Appendix

McGill Illness Narrative Interview (Danielle Groleau, Allan Young, & Laurence J. Kirmayer ©2006) adapted to explore the meaning and experience of FGM/C (Danielle Groleau ©2021).

### Section 1: narrative of FGM/C (The generic term FGM/C will be replaced by the local terminology)

1. When did you first hear about the possibility that you might go through FGM/C [*Let the narrative go on as long as possible, with only simple prompting by asking*, ‘What happened then? And then?’]
2. We would like to know more about your experience. Could you tell us how you felt when you became conscious that this will most likely happen to you?
3. Can you tell me the story of what happened when you underwent FGM/C? (Who did it, context, other girls at the same time, what was cut, any medication)
4. Did something else happen? [*Repeat as needed to draw out contiguous experiences and events. This can include information on ritual context, other girls cut at the same time, care or help from family, or that they participated in the procedures*]
5. Did you experience any difficulties after the procedure?
  If yes, could you tell me the story of how these came about?
  5.1 Did you seek any help or discuss with someone any of these difficulties or concerns?
  5.2 If so, did you find this supportive?
  5.3 What happened afterwards.
6. Did you have any treatments because of complications related to your FGM/C?
  6.1 If yes, please tell us how this happened?

### Section 2: prototype narratives

7. In the past, have you ever been concerned or worried about another bodily experience or health issue that you consider similar to your experience with FGM/C? [If answer to #7 is Yes, then ask Q.7.1.]
  7.1 In what way where these experiences similar or different from your experience with FGM/C?
8. Did a person in your family have FGM/C? Who? [If answer to #8 is Yes, then ask Q.8.1.]
  8.1 In what ways do you consider your experiences of FGM/C to be similar to or different from this other person’s experiences?
9. Did a person in your social environment (friends or work) experience FGM/C? [If answer to #9 is Yes, then ask Q.9.1.]
  9.1 In what ways do you consider your experience with FGM/C to be similar to or different from this other person’s experience?
10. Have you ever seen, read or heard on television, radio, in a magazine, a book or on the Internet of a person who had similar concerns and experiences with FGM/C as you? [If answer to #10 is Yes, then ask Q.10.1]
  10.1 In what ways is that person’s concerns and experiences similar to or different from yours?

### Section 3: explanatory model narrative

11. Do you have another term or expression that describes FGM/C?
12. According to you, what caused you to undergo FGM/C? [List primary cause(s).]
13. Are there any other causes that you think played a role? [List secondary causes.]
14. Why was your FGM/C arranged when it did?
15. Do you think this was a good time for it to happen?
16. Was there something happening in your family, at work or in your social life that has affected the way you feel about your FGM/C?
17. Do you think most girls/women your age in your community also have undergone FGM/C?
18. What do you think is the difference between those who have, and those who have not undergone FGM/C in your community?
19. What usually happens to those who have not had FGM/C?
20. Is there a way to name or label someone who experiences emotional or psychological difficulties in relation to FGM/C?
21. What is the best way to help solve this type of problem?
22. Can you tell me the story of how you felt before, during and after you underwent FGM/C?
23. Did you have worries or troubles related to FGM/C?
24. Do you think this relates to other events or periods in your life?

### Section 4: services and response to treatment

25. Have you have ever sought help for information concerning worries, or emotional or other difficulties that might be related to your FGM/C?
26. Did the person from whom you sought help give you any treatment, medicine or recommendations to follow for these difficulties? [List all]
27. How are you dealing with each of these recommendations? [*Repeat Q. 27.1 to Q. 27.4. as needed for every recommendation, medicine and treatment listed by interviewee.*]
  27.1 Were you able to follow that treatment (or recommendation or medicine)?
  27.2 What made that treatment work well?
  27.3 What made that treatment difficult to follow or work poorly?
  27.4 What treatments did you expect to receive for your difficulties that you did not receive?

### Section 5: impact on life and identity

28. How has your FGM/C change the way you experience life?
29. How has the emotional or other difficulties you experienced in relation with FGM/C change the way you experience life?
30. How has FGM/C change the way others look at you?
31. Could you tell us if FGM/C has impacted your intimate or sexual relationships?
32. What has helped you overcome these difficulties?
33. How has your faith, spiritual or religious practice helped you go through this period of your life?
34. Would you like to add anything else regarding your experiences or difficulties in relation to FGM/C?

## Abbreviations

FGM/C: Female genital mutilation/cutting
UNICEF: United Nations Children’s Fund
WHO: World Health Organization

## Footnotes

1 A 5 day training workshop with theory and practice sessions on qualitative research methods, qualitative interviewing as well as on the McGill Illness Narrative Interview (MINI) was given to the team by Danielle Groleau. Supervised practice sessions were held with the research team interviewing women of Igbo origin.
